# Elucidating Adverse Nutritional Implications of Exposure to Endocrine-Disrupting Chemicals and Mycotoxins through Stable Isotope Techniques

**DOI:** 10.3390/nu10040401

**Published:** 2018-03-23

**Authors:** Victor O. Owino, Carolin Cornelius, Cornelia U. Loechl

**Affiliations:** Nutrition and Health Related Environmental Studies Section, Division of Health, International Atomic Energy Agency, Vienna International Centre P.O. Box 100, A-1400 Vienna, Austria; Carolin.Cornelius@meduniwien.ac.at (C.C.); C.U.Loechl@iaea.org (C.U.L.)

**Keywords:** endocrine disruptors, aflatoxins, food and environmental hazards, child growth and development, stable isotope techniques

## Abstract

Multiple drivers of the double burden of malnutrition (DBM) include a rapid shift from predominantly plant-based diets to energy-dense foods based on meats, milk, animal fats and vegetable oils. The shift to overweight and obesity is driven by increased exposure to mass media, urbanization, technological advances in food processing, rising income and increased population density associated with increased access to cheap foods. At the same time, undernutrition persists mainly due to food insecurity and lack of access to safe water, sanitation and adequate health care. All known nutrition interventions result in only one third reduction in stunting. Little consideration has been given to hazardous exposure to endocrine disrupting chemicals (EDCs) and microbial toxins as major components of the malnutrition-causal framework. These hazards include microbial toxins, for example, mycotoxins, and environmental pollutants such as persistent organic pollutants (POPs), some of which are known to disrupt the endocrine system. These hazards sit at the cross road of undernutrition and overweight and obesity since the exposure cuts across the critical window of opportunity (the first 1000 days). In this review, we update on the role of food and environmental contaminants, especially EDCs and aflatoxins, in child growth and on the implications for metabolic dysfunction and disease risk in later life, and discuss potential applications of nuclear and isotopic techniques to elucidate the underlying biological mechanisms, outcome indicators, as well as occurrence levels.

## 1. Introduction

The United Nations General Assembly proclaimed the Decade of Action on Nutrition on 1 April 2016 in response to the urgent need to reverse the increasing trend of the number of people globally affected by the double burden of malnutrition characterised by co-existence of chronic undernutrition and micronutrient deficiencies on the one hand and overweight and obesity on the other. The double burden of malnutrition (DBM) affects at least 40% of countries worldwide [1]. Stunting remains a major global problem with 159 million children under the age of 5 years being affected. At the same time, a total of 41 million children suffer from overweight [1]. Growth retardation in the first 1000 days is linked to risk for overweight, obesity and related non-communicable diseases (NCDs) in later childhood, adolescence and adulthood [2]. All these conditions lead to reduced human potential.

The Decade of Action on Nutrition recognizes the emergence of local, national regional and global movements to end all forms of malnutrition [3]. All countries will be supported to address all forms of malnutrition and their causes, including food safety as a key component.

In order to achieve the objectives of the Decade of Action on Nutrition and the global targets on nutrition by 2025 and 2030, respectively, the global community must have a broader sense of what drives the DBM and associated adverse health effects. The DBM has multiple drivers including a rapid shift from predominantly plant-based diets to energy-dense foods based on meats, milk, animal fats and vegetable oils [4]. Low and middle income countries (LMICs), defined based on the World Bank Atlas Method as having a Gross National Income (GNI) of between US$1005 and US$3955 or less in 2016 [5]. Major drivers of the shift to overweight and obesity in LMICs have been identified as increased exposure to mass media due to globalisation, urbanization, technological advances in food processing, rising income and increased population density associated with increased access to cheap foods [6]. At the same time undernutrition persists mainly due to food insecurity and lack of access to safe water, sanitation and adequate health care. Evidence shows that all known nutrition interventions result in only one third reduction in stunting [7]. Nutrient deficiencies remain major causes of malnutrition. More recently, living in an unsanitary environment has been recognised as one potent factor in growth retardation [8,9,10].

Little consideration has been given to hazardous exposure to endocrine disrupting chemicals and microbial toxins as a major component of the malnutrition-causal framework. These hazards include microbial toxins, for example, mycotoxins, and environmental pollutants such as persistent organic pollutants (POPs), some of which are known to disrupt the endocrine system, amongst others. These hazards sit at the cross road of undernutrition and overweight and obesity since the exposure cuts across the critical window of opportunity (the first 1000 days) from pregnancy to the first 2 years of life.

Accumulating evidence shows that exposure to numerous chemicals, both natural and man-made, may have an impact on the endocrine system, with far-reaching health implications in humans and wildlife [11]. Scientists often refer to these chemicals as endocrine-disrupting chemicals (EDCs). The Endocrine Society defines an endocrine disruptor as “an exogenous chemical, or mixture of chemicals, that can interfere with any aspect of hormone action” [12,13]. Despite the growing body of evidence on their potential adverse effect on child health, such contaminants have received less attention from the nutrition community. Mycotoxins, especially aflatoxins, have been strongly linked to child growth retardation [14,15]. Although some POPs are recognised as endocrine disruptors with a potential to affect child growth and health, they have received little attention.

**Figure 1:**
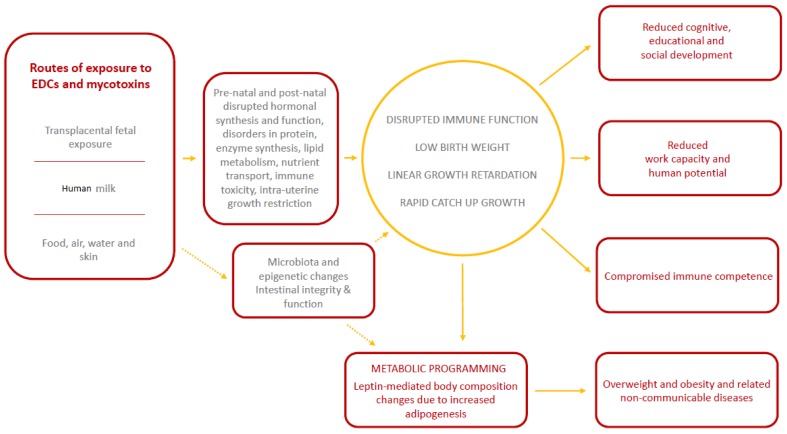
Conceptual framework for the effects of exposure to endocrine disrupting chemicals (EDCs) and mycotoxins on pre-natal and post-natal growth and long-term health and development outcomes.

Although, the biological pathways through which EDCs and mycotoxins may affect child growth and development are not well defined, prenatal and postnatal events including disrupted hormonal synthesis and function, disorders in protein, enzyme synthesis, lipid metabolism, nutrient transport, immune modulation and toxicity which may partly lead to intra-uterine growth restriction and ultimate low birth weight and postnatal growth retardation [16,17,18,19,20,21,22] are thought to be involved (Figure 1). A recent review suggested that the exposure to EDCs results in genetic and gut microbiota changes [23]. Whether mycotoxins have similar anti-biotic effects in humans remains to be explored since the only available evidence is based on animal studies [24]. More information is required to enhance the body of knowledge regarding the pathways through which food and environmental contaminants affect human health, the levels of occurrence in foods and the environment, and establishment of associations between occurrence, exposure and effects. A better understanding of exposure to potentially unsafe levels of multiple classes of EDCs is required to support nutrition and public health programmes and authorities. In this review, we update on the role of food and environmental contaminants, especially EDCs, in child growth and implications for disease risk in later life, and discuss potential applications (or potential use) of nuclear and isotopic techniques to elucidate the underlying mechanisms, as well as occurrence levels.

Exposure to EDCs and mycotoxins likely leads to growth retardation through foetal programming occasioned by disturbed hormonal, enzyme and nutrient synthesis, transport and metabolism and immune modulation resulting in intrauterine growth restriction and eventually low birthweight. Rapid catch up growth linked to low birth weight then leads to metabolic programming favouring excess adiposity eventually resulting in overweight and obesity. Changes in microbiota composition and function and gene alterations may also be involved but more research needs to be done in humans. Dotted lines indicate areas where more research is needed in future.

## 2. Endocrine Disruptors

Adverse health effects associated with EDCs include abnormal growth patterns, male and female reproductive abnormalities, neurobehavioral deficits, metabolic syndrome, bone disorders, immune disorders and some types of cancer [12].

EDCs represent a broad class of molecules which affect various endocrine functions, including hormonal synthesis and transport in a dose-dependent manner [25]. While many of the EDCs are produced for distinct purposes and are found in various materials, such as organochlorinated pesticides, industrial and medical chemicals, plastics, metals, personal care products (e.g., perfumes or cosmetics), electrical transformers and many other products [11,25], some of the EDCs occur only as by-products during manufacturing processes or emerge as breakdown products of other chemical compounds. Some, such as ethinylestradiol and diethylstilbestrol, are synthetic human or animal drugs, while others are found in nature, such as flavonoids found in fruits and vegetables [26,27].

Human exposure to EDCs occurs by a number of mechanisms, including ingestion of contaminated food or water, inhalation of gases and particles, through dermal absorption, through invasive medical instruments or during pregnancy. Due to the consumption of more calories per body surface and a higher minute ventilation compared to adults, children may be at higher risk of exposure to EDCs than adults [28].

Furthermore, food and environmental contaminants can be transferred from a pregnant woman to the foetus or neonate through the placenta or via breastfeeding. Pregnant women and children are the most vulnerable groups to exposure to EDCs. Moreover, the developmental consequences of exposure may often not be apparent until later in life [26,29].

Key Messages:

“A holistic consideration of all potential drivers of the double burden of malnutrition will maximize the desired results of the Decade of Action on Nutrition and the achievement of the global nutrition targets. Despite accumulating evidence on their potential adverse effect on child health, environmental contaminants have received less attention from the nutrition community.”

“Food and environmental contaminants may modify child growth bi-directionally, either leading to retardation or undesirably rapid height gain related to increased energy efficiency. Endocrine disruptors are also associated with increased fasting insulin, increased body mass index and reduced cognitive and neuro-development. Mycotoxins have been strongly linked to stunting and immune function impairment.”

“Nuclear techniques, especially stable isotope techniques have been applied for decades to understand biological pathways underpinning human health including assessment of body composition, energy expenditure, nutrient absorption and bioavailability in addition to assessment of infant and young child feeding practices. Nevertheless, these techniques have so far not found use in the understanding of the link between POPs and child growth and later health.”

“Current methods to characterize exposure to POPS from dietary sources are inaccurate. The underlying biological pathways linking POPs and aflatoxins to child growth metabolic dysfunction are not fully understood.”

“Nuclear and stable isotope techniques can be applied for characterization of exposure and understanding physiological and metabolic changes such as body composition, energy balance and physical activity associated with these exposures, as well as determining concentrations in food and environmental matrices.”

### 2.1. The Link between Endocrine Disruptors and Pre-Natal and Early Childhood Growth and Long-Term Health and Development

So far, little is known about the association between the exposure to EDCs and child growth. However, accumulating evidence indicates that prenatal or early-life exposure to EDCs has the capability to adversely affect foetal as well as later growth in children [19,20,21,25,28]. Given the importance of hormones in tissue differentiation, the developing child is very vulnerable to levels and fluctuations of chemicals with hormonal or anti-hormonal activity such as EDCs [30]. For instance, production of abnormal levels (high or low) of the growth hormone by the pituitary gland due to interference with EDCs, may result in a child growing excessively tall or abnormally short [31]. Furthermore, exposure to environmental chemicals may result in hypothyroidism, a condition related to very low levels of thyroid hormones in the blood, which may lead to slower growth and delayed puberty. Exposure to EDCs is also linked to precocious puberty (pubertal development before the age of 8 years in a girl or 9 years in a boy) [32] and reduced adult stature [31,33]. A large European study assessing the effect of maternal occupational exposure to EDCs showed an increased risk of term low birth weight (LBW) among children of exposed mothers [34]. LBW is not only a determinant of short-term linear growth and survival but it is also a risk factor for later metabolic dysfunction. Endocrine disruptors may limit the function of growth promoting hormones (for example, insulin, inulin growth factors 1 and 2) while aiding the function of growth-inhibiting ones such as glucocorticoids [35].

### 2.2. The Potential Association between Endocrine Disruptors and Metabolic Dysfunction

Although the underlying mechanisms between EDCs and metabolic dysfunction have not yet been fully elucidated, there is growing evidence that EDCs may interfere with epigenetic, structural, and functional pathways which are involved in the regulation of lipid metabolism and adipogenesis [16,36]. This interference is thought to happen via a leptin-mediated activation of adipose depots with the release of a variety of hormones and cytokines [37]. These metabolic changes are likely to increase pre-disposition to obesity.

Obesogens, including EDCs, may interfere with critical metabolic signalling, for example nuclear hormone receptors, particularly the peroxisome proliferator-activated receptors (PPARα, δ and γ), which play important roles in the transcriptional control of lipid metabolism and adipogenesis [38,39], leptin-mediated changes in adiposity and inflammation [25,39,40].

Prenatal exposure to polychlorinated biphenyls (PCBs) and *p*,*p*’-dichlorodiphenyldichloroethylene (DDE) has been linked to increased body mass index (BMI), waist circumference and change in BMI from 5 to 7 years of age among girls with overweight mothers [41] as well as high fasting insulin levels among girls [42]. This effect was thought to be due to an early onset of adiposity rebound. A recent review of the link between EDCs and obesity observed that prenatal exposure to some of the chemicals lead to decreased birth weight which is in turn linked to disproportionately increased adipose mass after puberty [25]. As such, EDCs may be involved in foetal programming through hormonal changes that impact birth weight and later risk for non-communicable diseases [35]. Indeed, a study from India demonstrated that children born small for gestational age were insulin resistant and that poor intrauterine growth predicted higher central adiposity at 8 years of age [43]. Insulin resistance associated with intrauterine growth restriction was linkedwith postanatal catch-up growth in France [18].

### 2.3. The Link between Mycotoxins and Child Growth

Aflatoxins are secondary metabolites produced by moulds, especially *Aspergillus flavus* and *Aspergillus parasiticus* [44]. Aflatoxin B1 (AFB1) is the most frequently occurring and the most toxic and carcinogenic, whilst other members of the family include AFB2, AFG1, and AFG2. Aflatoxin occurs in staple foods, especially cereals and legumes. Maize and groundnuts are most vulnerable. Per capita maize consumption is directly correlated to the risk of aflatoxin exposure and to some extent to the geographical distribution of stunting [45]. Furthermore, numerous studies have demonstrated adverse groath outcomes among children consuming maize contaminated with aflatoxins [46,47,48].

Infants may be exposed to aflatoxin via maternal food intake in utero [21], via breast milk, and via complementary or family foods [49]. Enhanced aflatoxin exposure in children is strongly associated with the introduction of family foods [50].

Commonly used biomarkers for aflatoxin exposure are aflatoxin albumin adduct in serum and aflatoxin M1, a less toxic metabolite of AFB1, in body fluids such as urine and breast milk [44,51]. Aflatoxin exposure has been linked to impaired growth [14,22] and kwashiorkor [50], and may also have a role in the modification of the aetiology of hepatitis B [52,53] in African children. A longitudinal study from Benin assessed the association between aflatoxin exposure and growth, vitamin A and zinc plasma levels in 200 children 16–37 months of age [14]. Children with the highest quartile of aflatoxin concentration in albumin had a mean of 1.7 cm reduction in height over 8 months compared with those at the lowest quartile.

It is possible that aflatoxin exposure in infants may exacerbate the adverse effects of HIV exposure such as growth faltering [21] and immunological deficiencies [54,55]. A cohort study of 472 Gambian children aged 6–9 years old suggested an association between dietary aflatoxin exposure and reduced secretory IgA in saliva [22]. Aflatoxin exposure may also be linked to intestinal inflammation related to growth hormone (GH) resistance recently demonstrated in a rat model. Stunted children in low- and middle-income countries are thought to suffer from environmental enteric dysfunction (EED), also related to intestinal villous blunting, inflammation and absorptive ad barrier dysfunction [9,10].

Although the mechanisms by which aflatoxin exposure affects infant growth are not well understood, the possibilities include: immune function modification, disorders in protein and enzyme synthesis and lipid metabolism; this may be associated with changes in body composition (fat-free and fat mass accrual) and changes in intestinal integrity down-regulation of genes associated with energy production and fatty acid metabolism [22].

## 3. The Role of Nuclear/Isotopic Techniques in Characterisation of Food and Environmental Hazards and Understanding Pathways through Which They Affect Human Health

Nuclear techniques, especially stable isotope techniques have been applied for decades to help in understanding the biological pathways underpinning human health including assessment of body composition, energy expenditure, nutrient absorption and bioavailability in addition to assessment of infant and young child feeding practices [56]. Stable isotope techniques have also been applied in the detection of food contaminants including arsenic [57] and mycotoxins [58]. Despite their wide application, nuclear techniques have so far not found use in elaborating the link between POPs with endocrine disrupting activity and child growth and later health. The application of nuclear and isotopic techniques offers a number of opportunities to investigate and increase knowledge and understanding of these issues including: (1) detection of the hazards in food and related matrix, (2) characterisation of the risk (risk from breast milk is used as an example here), and (3) assessment of indicators of metabolic function modifiable by exposure to the hazards.

### Characterising Exposure to EDCs from Human Milk

Breast milk is the best food for infants; breastfeeding reduces child mortality and is linked to improved IQ [59]. However, POPs contain a variety of lipophilic components, that often resist biological and chemical degradation and therefore preferentially bio-accumulate in lipid-containing tissues [60] and human milk and are transferred from the mother to the infant during breastfeeding [60]. Characterisation of exposure to POPs in breast milk involves three key tenets: determination of the amount of breast milk consumed by the infant, measurement of human milk lipid content and analysis of POPs in the milk lipid fraction.

Numerous studies have reported the estimation of exposure to POPs from breast milk [61]. The limitation in all these studies is that human milk intake was based on estimation or by manual expression of milk by the mother [61]. Cross sectional manual expression of human milk may not depict habitual consumption by the infant. The ability to quantify exposure to POPs from breast milk can be enhanced by using a safe, objective and non-invasive stable isotope technique that measures human milk intake over a two-week period, the deuterium oxide dose-to-mother technique [56,62]. A lactating mother drinks a dose of deuterium oxide (D_2_O), which is distributed throughout her body within a few hours and is incorporated into her milk. The baby receives deuterium only during breastfeeding. The saliva and urine of both the mother and child is enriched with deuterium and this can be measured using a Fourier Transform Infrared Spectrometer (FTIR) or an Isotope Ratio Mass Spectrometer (IRMS) [62]. Saliva samples are collected from both the mother and child over a period of 14 days. Deuterium enrichment in saliva compared to a baseline is then used to calculate the amount of breast milk consumed by the infant.

## 4. Assessment of Metabolic Changes Linked to Exposure to EDCs

### 4.1. Body Composition

Body composition refers to the components into which the body is divided. The assessment of body composition can be considered as an indicator of nutritional status and healthy growth [46]. Body composition changes are sensitive to environmental factors and would respond to the hormonal and metabolic changes associated with exposure to EDCs. Body fat accumulation and distribution is an important indicator for assessing the risk for overweight, obesity and related NCDs. Excess body fat is associated with physiological changes that can lead to NCDs, such as diabetes, cardiovascular disease and some cancers. On the other hand, excess accumulation of visceral fat is also associated with higher risk for NCDs. A good example of this is the ‘thin-fat-Indian baby phenomenon’ described elsewhere [63].

Tracking of fat mass can be a very good indicator of how exposure to EDCs may affect the body’s metabolic function. The deuterium dilution technique has been applied over time to accurately measure fat mass and fat-free mass (FFM) in different contexts. In this technique, a pre-dose saliva sample is collected from a person before she or he drinks an accurately weighed amount of D_2_O and a post-dose saliva sample is collected 3–5 h later. Based on the D_2_O enrichment in saliva, FFM can be determined by measuring total body water (TBW) and then using an appropriate hydration factor to calculate FFM [64]. Fat mass is the difference between FFM and total body weight. Body fat distribution can be assessed using Dual Energy X ray Absorptiometry (DXA) which is also useful for determination of bone mineral density [65].

### 4.2. Nutrient Absorption

The proportion of nutrients absorbed and retained by the body for function is a critical determinant of child growth and development. No study has reported on how EDCs may affect the absorption of nutrients, especially of amino acids, iron, and zinc, for example. Existing isotopic techniques that can assess nutrient absorption are therefore likely to be of immense use in contributing much needed data in this area. Stable isotopes have been applied widely to measure the absorption and bioavailability of iron, zinc, and vitamin A. The development of a method to assess the digestion and bioavailability of proteins and amino acids from food legumes intrinsically labelled with stable isotopes is at an advanced stage ([56], Preston et al. Unpublished).

### 4.3. Energy Expenditure and Physical Activity

To our knowledge, no study has investigated how exposure to EDCs may influence energy balance and physical activity in children. Physical inactivity is a major risk factor for NCDs. This is an area where stable isotopes may add value. Doubly labelled water (DLW) with both deuterium (^2^H_2_O) and oxygen-18 (H_2_^18^O) has been widely used to assess habitual energy expenditure over a time-period of 7–20 days. This enables the calculation of the level of individual physical activity [56,66]. An individual is given orally an accurately weighed dose of DLW, which is distributed throughout their body water. During normal breathing or exercise, some of the labelled oxygen and hydrogen is lost in urine, sweat, and breath. Deuterium is lost only in water, whereas oxygen-18 is lost in both water and carbon dioxide. The difference in the elimination rates of deuterium and oxygen-18 is a measure of carbon dioxide production rate, from which energy expenditure can be calculated after analysing urine samples using an isotope ratio mass spectrometer [56,66].

## 5. Conclusions

Persistent organic pollutants, including those that are endocrine disruptors, affect individual nutritional status and risk for NCDs in ways that are still not fully understood. POPs have been linked to increased body mass index, higher fasting insulin levels and impaired cognitive and neuro-development. To confirm these effects, accurate methods are needed to characterise exposure to POPs from the food chain and the environment, and to better understand the affected biological pathways, how these hazards affect growth and development, and how they increase the risk for NCDs. How EDCs and mycotoxins affect gut microbiota and epigenetics and how these link with child growth and risk for metabolic dysfunction remains to be further explored.

There is an urgent need to establish closer linkages and corroboration between food safety and nutrition matters, as well as attendant end points such as growth retardation, amongst others. To realize this, a stronger laboratory testing and monitoring culture has to be established in Member States, to accurately determine prevalence and occurrence levels of the various potential and known EDCs. This requires robust and sensitive analytical techniques that provide for a high degree of precision.

Dietary exposure to various hazards of concern to nutrition (such as aflatoxins) may be determined using the aforementioned analytical techniques, and this may be alongside dietary intake surveys [15]. To better correlate the hazards and attendant toxic effects, knowledge of biomarkers is required as accurate indicators of exposure and internal effective dose [15]. Nuclear and isotopic techniques can help address this challenge and contribute to a better understanding of EDCs and aflatoxins, and their effect on child growth retardation and other associated end points. Techniques such as radio-immuno assays, radio receptor assays, and various stable isotopes used along with mass spectrometry are applied routinely to determine and monitor food and environmental contaminants, including POPs. The same may be tailored in particular to EDCs. The deuterium dilution technique is useful in characterising risk from breast milk and in assessing body composition as an indicator of metabolic changes.
